# In vitro and in vivo consequences of variant medium-chain acyl-CoA dehydrogenase genotypes

**DOI:** 10.1186/1750-1172-8-43

**Published:** 2013-03-20

**Authors:** Catharina ML Touw, G Peter A Smit, Klary E Niezen-Koning, Conny Bosgraaf-de Boer, Albert Gerding, Dirk-Jan Reijngoud, Terry GJ Derks

**Affiliations:** 1 Section of Metabolic Diseases, Beatrix Children’s Hospital, University of Groningen, University Medical Centre, Groningen, The Netherlands; 2 Laboratory of Metabolic Diseases, Department of Laboratory Medicine, University of Groningen, University Medical Centre, Groningen, The Netherlands; 3 Center for Liver, Digestive and Metabolic Diseases, University of Groningen, University Medical Centre of Groningen, CA84, PO Box 30 001, Groningen 9700 RB, The Netherlands

**Keywords:** ACADM, Enzyme, Genotype, Fasting, Phenylpropionic acid

## Abstract

**Background:**

Medium-chain acyl-CoA dehydrogenase (MCAD) deficiency is the most common inherited disorder of the mitochondrial fatty acid oxidation, caused by mutations in the *ACADM* gene. Since the introduction of neonatal screening for MCAD deficiency, a subgroup of newborns have been identified with variant *ACADM* genotypes that had never been identified before in clinically ascertained patients. *In vitro* residual MCAD enzyme activity has been found to facilitate risk-stratification. In this study we integrated results of *in vitro* (residual MCAD enzyme activities) and *in vivo* (clinical fasting tolerance tests, and phenylpropionic acid loading tests) tests in this subgroup of newborns, defining the consequences of variant *ACADM* genotypes.

**Methods:**

Enzyme analyses were performed in leukocytes with: hexanoyl-CoA (C6-CoA) +/− butyryl-CoA (C4-CoA), and phenylpropionyl-CoA (PP-CoA). *In vitro* studies were performed in 9 subjects with variant *ACADM* genotypes, *in vivo* functional tests in 6 of these subjects.

**Results:**

Enzyme analyses with C6-CoA, C6-CoA + C4-CoA, and PP-CoA identified significantly higher residual MCAD enzyme activities in subjects with variant *ACADM* genotypes when compared to patients with classical *ACADM* genotypes.

After prolonged fasting (range 15–18.5 hours) no hypoglycaemia was observed. Increasing concentrations of free fatty acids indicated lipolysis, and ketone body concentrations were sufficient for blood glucose concentrations in 5 out of 6 subjects. Phenylpropionic acid loading clearly demonstrated *in vivo* residual MCAD enzyme activity in all studied subjects.

**Conclusions:**

Subjects with variant *ACADM* genotypes and residual MCAD enzyme activities >10% display residual MCAD enzyme activities *in vitro* and *in vivo*. Our findings support the hypothesis that the guidelines on maximal duration of fasting might be abandoned in subjects with residual MCAD enzyme activities >10% under normal conditions. An emergency regimen and parental instructions remain necessary in all subjects with MCAD deficiency, regardless of residual MCAD enzyme activity.

## Introduction

Inherited disorders of mitochondrial fatty acid oxidation (mFAO) are a group of acute presenting, life-threatening disorders among which medium-chain acyl coenzyme A dehydrogenase (MCAD [E.C.1.3.99.3; OMIM 201450]) deficiency is the most common [1]. Worldwide, population neonatal bloodspot screening (NBS) programs have become available for the disorder.

Before the introduction of MCAD deficiency in NBS programs, patients presented clinically with symptoms associated with a life-threatening hypoketotic hypoglycaemia, such as seizures, coma or even sudden death. The c.985A>G missense mutation on the *ACADM* gene (gene encoding MCAD, OMIM 607008) was the most common mutation. Since the introduction in NBS programs, the spectrum of *ACADM* genotypes changed dramatically. Novel *ACADM* genotypes were identified in prospectively screened asymptomatic newborns (i.e. variant *ACADM* genotypes), of which the clinical consequences are currently unknown. In previous studies our group and others emphasized the importance of determi-nation of residual MCAD enzyme activity, both for diagnostic and prognostic purposes [2,3]. However, studies integrating laboratory (*in vitro)* and clinical (*in vivo)* data are scarce [4].

Currently, a late evening meal is advised during the first two years of life for patients with MCAD deficiency, regardless of the *ACADM* genotype [2]. Based on *in vitro* data, the necessity to treat all subjects with MCAD deficiency similarly is debatable, in particular with regards to the advice on maximum duration of fasting [2]. However, *in vivo* fasting tolerance under controlled conditions has not been studied before in this group of subjects. It can be hypothesised that residual MCAD enzyme activities and clinical fasting tolerance tests reflect the *in vitro* and *in vivo* role of these variant *ACADM* genotypes. In order to enable personalized care after diagnosis, clinical fasting tolerance tests were performed in our centre in subjects with variant *ACADM* genotypes after informed consent of the parents. In this study, we integrated data on *in vitro* MCAD enzyme assays, and *in vivo* functional tests from 9 subjects with variant *ACADM* genotypes.

## Methods

The Medical Ethical Committee of the University Medical Centre Groningen approved the study (METc 2011/133). Parents agreed on participation by written informed consent.

### Cohort

In the Netherlands MCAD deficiency has been included in the national population NBS program since 2007. Free carnitine (C0), octanoylcarnitine (C8:0), decanoylcarnitine (C10:0), and the C8:0/C10:0 ratio are determined in dried blood spots obtained from newborns 72 to 168 hours after birth (http://www.rivm.nl). Clinical and laboratory follow-up is initiated within 24 hours in newborns in case of elevated C8:0 concentrations. During the pilot NBS program in 2003–2006 the cut-off concentration for C8:0 was 0.30 μmol/l [5]. Since 2007, the cut-off concentration for C8:0 has been ≥ 0.50 μmol/l [2]. Diagnosis of MCAD deficiency is made after abnormal NBS, based on persisting abnormal metabolite profiles, the presence of 2 mutations on the *ACADM* gene, and/or residual MCAD enzyme activity <50% when measured with hexanoyl-CoA (C6-CoA) in leukocytes. Laboratory analysis is always performed in family members of a proband, including all siblings.

Subjects carrying ‘variant *ACADM* genotypes’, who were diagnosed at the Beatrix Children’s Hospital, UMC Groningen, Groningen, The Netherlands between 2003–2011 were included in our cohort. A ‘variant *ACADM* genotype’ was defined as an *ACADM* genotype that had not been described before in clinically ascertained patients in either The Netherlands [6] or in literature.

Maximum percentage of weight loss was calculated as the ratio between the birth weight and the lowest weight observed in the first neonatal period.

### Enzyme analysis

Residual MCAD enzyme activity was determined in leukocytes. Before 2007, a GC-MS based analysis using C6-CoA +/− butyryl-CoA (C4-CoA) as substrates was the standard in our centre [7,8]. The assay with C6-CoA has been described extensively, and was modified for more accurate analysis of MCAD enzyme activity and elimination of the contribution of short-chain acyl-CoA dehydrogenase (SCAD; OMIM 606885). In this assay 785.7 μM C4-CoA was added as a substrate, besides C6-CoA. At least one commercially available leukocyte pellet (Sanquin, The Netherlands) was used as control in each enzyme assay. Molecular analysis of the *ACADM* gene was performed in all subjects with residual MCAD activity <50% when determined with C6-CoA. Since 2007, an HPLC-based assay using 3-phenylpropionyl-CoA (PP-CoA) as a substrate is used [5,9]. Residual enzyme activities are expressed as a percentage from healthy controls.

### Clinical fasting tolerance tests

All clinical fasting tolerance tests were performed in the clinical function test ward of the Beatrix Children’s Hospital, UMC Groningen, according to established protocols [10,11]. Hypoglycemia was defined as blood glucose concentrations <2.6 mmol/l [12]. The fasting tolerance test was combined with a 3-phenylpropionic acid (PPA) loading test, where 25 mg/kg PPA was administered orally [13]. Two portions of urine were collected during the fasting tolerance test. Portion 1 was obtained after overnight fasting in the first 4 hours following PPA loading; the second portion was obtained in the following 4 hours of the fasting tolerance test. All PPA loading tests were performed after the age of 6 months [14]. Urinary phenylpropionylglycine was determined by gas-chromatography-mass spectrometry (GC-MS), according to Chalmers *et al.*[15]*.*

### Data analysis

Clinical and laboratory data, and data from functional tests were retrospectively retrieved from the medical charts and laboratory files of the subjects by one investigator (CT).

Differences between normally distributed continuous data were analysed using parametric tests. Data that were not normally distributed were analysed using nonparametric tests. For analysis of correlations, Spearman’s rank test was used. The significance level was set at p < 0.05. Statistical analyses were performed using GraphPad Prism software (GraphPad Software Inc., version 5.00, 2007).

## Results

### Cohort

From the birth cohort 2003–2011, 50 newborns were diagnosed with MCAD deficiency in our centre after referral for a positive NBS (in either the pilot screening program, or the regular nationwide NBS). In 9 of these 50 subjects (18%) a variant *ACADM* genotype was identified (Table 1). In two of these subjects (cases 8 & 9), only one *ACADM* mutation was detected after sequencing of all exons and adjacent introns. However, acylcarnitine profiles in these subjects were indicative of MCAD deficiency, and residual MCAD enzyme activities were <50% when measured with C6-CoA.

**Table 1:** **Characteristics of subjects with variant *ACADM *genotypes during fasting tolerance tests and PPA loading tests**

**RESULTS FASTING TEST AND PPA LOADING TEST IN CHILDREN WITH VARIANT *ACADM *GENOTYPES**																	
**Case**	**Genotype**		**NBS**			**MCAD activity (%)**			**Fasting test**								**PPA loading test**
	**Allele 1**	**Allele 2**	**C8:0**	**C8:0/C10:0**	**Organic acids**	**C6-CoA**	**+C4-CoA**	**PP-CoA**	**Age (mo)**	**Duration (h)**	**Glc(t = 15 h)**	**KB(t = 15 h)**	Glc*KB(t = 15 h)	**FFA/KB(t = 15 h)**	**C8:0 (t = 15 h)**	**C8:0/C10:0**	**PP-glycine**
**1**	c.985A>G	c.199T>C	2.85	2.3	Normal	39	36	-	-	-	-	-	-	-	-	-	-
**2**	c.985A>G	c.199T>C	0.67	2.7	DC, HG	44	29	58	-	-	-	-	-	-	-	-	-
**3**	c.985A>G	c.199T>C	0.35	2.3	Trace N-HG, SG, DC	48	43	48	-	-	-	-	-	-	-	-	-
**4**	c.985A>G	c.199T>C	0.74	2.8	Trace HG	36	34	44	10	18	4.5	1.1	5.0	1.0	3.7	3.4	Trace
**5**	c.985A>G	c.473A>G	0.39	2.3	Normal	38	25	39	6	18.5	2.8	0.3	0.8	2.2	2.0	3.8	Absent
**6**	c.985A>G	c.734C>T	1.41	3.0	Trace DC, 5-OH-C, HG, SG	41	17	11	13	17	4.4	0.8	3.3	1.9	3.6	4.2	Trace
**7**	c.985A>G	c.928G>A	2.28	3.2	5-OH-H, trace HG	48	23	0	16	15	3.2	3.5	10.6	-	1.9	2.9	Absent
**8**	c.985A>G	Not found	0.70	1.3	Normal	28	-	-	25	18.5	4.0	0.9	3.7	1.5	2.9	2.1	Trace
**9**	c.985A>G	Not found	0.50	0.8	Normal	15	11	86	27	16.5	2.9	1.4	5.8	0.8	0.5	2.2	Trace
*	Classical *ACADM* genotype		2.98	12.7	5-OH-H, HG, PPG, SG, DC	28	14	0	-	17-24	3.1	0.6	5.7	2.2	-	-	High
**#**	Control population		-	-	-	-	-	-	0-24	15	4.1(3.1-4.8)	1.3(0.4-3.2)	5.5(1.2-15.4)	1.1(0.7-2.4)	-	-	Absent
**#**	Control population		-	-	-	-	-	-	25-84	15	4.6(3.8-5.3)	0.5(0.1-1.7)	2.1(0.4-8.9)	1.8(0.8-6.4)	-	-	Absent

MCAD activity is determined in leukocytes. Residual MCAD enzyme activities are depicted as percentage from controls. At least one control was included in each enzyme assay. Ref, reference value; -, not determined; DC, dicarboxylic acids; Glc, glucose; HG, *N-*hexanoylglycine; SG, *N-*suberylglycine; PPG, phenylpropionylglycine; 5-OH-C, 5-OH-capronic acid; 5-OH-H, 5-OH-hexanoic acid; PPA, phenylpropionic acid; KB, ketone bodies; FFA, free fatty acids. * Characteristics of patients with classical *ACADM* genotypes during fasting according to Bonnefont et al. [10], median values are depicted. For NBS and enzymatic data median values from the UMCG cohort are depicted. ^#^ Reference values according to van Veen et al. [11] Median reference values are depicted. p10-p90 are indicated between brackets.

Two subjects (cases 3 & 5) were diagnosed with C8:0 concentrations below 0.50 μmol/l. Case 3 was the younger sister of case 2, carrying the c.985A>G/c.199T>C *ACADM* genotype. Case 5 was identified during the pilot screening program. Maximum percentage of weight loss in the neonatal phase in our cohort of subjects with variant *ACADM* genotypes correlated with C8:0 concentrations upon NBS (Spearman r −0.89, p < 0.05; median 5.9%; range 4.7-9.8%). In cases 3 and 5 the maximum percentage of weight loss was respectively 4.7% and 5.7%; in cases 8 and 9 with only one identified *ACADM* mutation it was respectively 7.0% and 5.9%. None of the subjects with a variant *ACADM* genotype presented clinically with hypoketotic hypoglycemia, either in the neonatal period or after diagnosis.

### In vitro studies – residual MCAD enzyme activities

**Figure 1:**
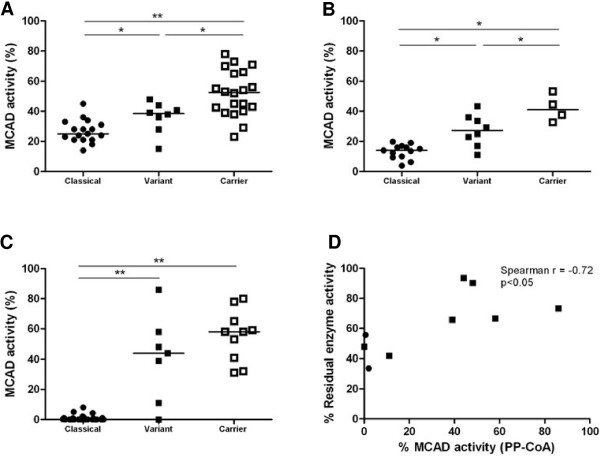
**Residual MCAD enzyme activities measured with different substrates in leukocytes. A**: Residual MCAD enzyme activities measured with C6-CoA; **B**: Residual MCAD enzyme activities measured with C6-CoA+C4-CoA; **C**: Residual MCAD enzyme activities measured with PP-CoA; **D**: MCAD enzyme activity with PP-CoA correlates with the effect of C4-CoA on C6-CoA enzyme activity. Enzyme activity measured with C6-CoA is set at 100% on the y-axis. Residual MCAD enzyme activities are depicted as percentage from controls. At least one control was included in each enzyme assay. Medians are indicated. * p<0.05; ** p<0.001.

**Figure 2:**
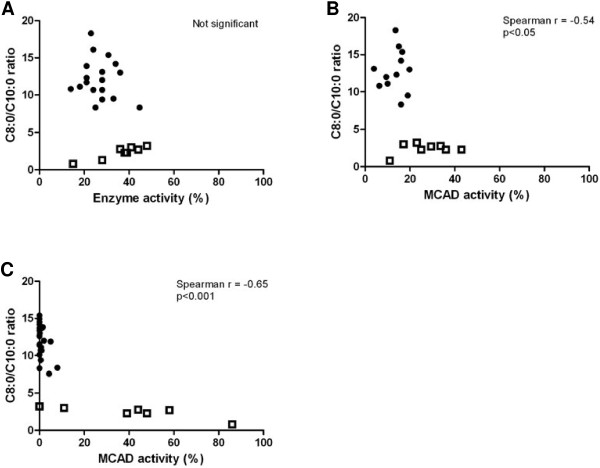
**Correlation between residual MCAD enzyme activities measured in leukocytes and C8:0/C10:0 upon NBS. A**: Residual MCAD enzyme activities measured with C6-CoA; **B**: Residual MCAD enzyme activities measured with C6-CoA+C4-CoA; **C**: Residual MCAD enzyme activities measured with PP-CoA. Residual MCAD enzyme activities are depicted as percentage of controls. At least one control was included in each enzyme assay. Variant ACADM genotypes are depicted as squares, classical ACADM genotypes as dots.

Results from *in vitro* analyses in the 9 subjects with variant *ACADM* genotypes are depicted in Table 1 and Figure 1. In 7 of these subjects MCAD enzyme analyses were performed in leukocytes with three different substrates: C6-CoA, C6-CoA + C4-CoA, and PP-CoA. Irrespective of the substrate, significantly higher residual MCAD enzyme activities were measured in subjects with variant *ACADM* genotypes, when compared to patients with classical *ACADM* genotypes (Figure 1). However, when measured with PP-CoA, case 7 demonstrated no residual MCAD enzyme activity. With the natural substrates C6-CoA and C6-CoA + C4-CoA, the group of subjects with variant *ACADM* genotypes differed clearly from both the group with classical *ACADM* genotypes, and from carriers of the c.985A>G mutation (i.e. siblings or parents of patients). The observed proportional change in enzyme activity after addition of C4-CoA to the C6-CoA assay correlated with the MCAD enzyme activity measured with PP-CoA (Spearman r −0.72, p < 0.05) (Figure 1D). Additionally, strong correlations were identified between residual MCAD enzyme activities measured with PP-CoA and C6-CoA + C4-CoA, and the C8:0/C10:0 ratio identified upon NBS (Spearman r −0.65; p < 0.001 and Spearman r −0.54; p < 0.01, respectively) (Figure 2).

### In vivo studies – fasting tolerance test

Clinical fasting tolerance tests were performed in 6 subjects with a variant *ACADM* genotype, at a median age of 14.5 months (range 6–27 months). The duration of fasting was supra-physiological in all subjects, as regular overnight fasting duration was extended. Case 7 fasted for 15 hours and the remaining 5 subjects for 16.5 – 18.5 hours (Table 1). Concentrations of glucose, free fatty acids (FFA), ketone bodies (KB), and their ratios after 15 hours of fasting are depicted in Table 1. All subjects could complete the fasting tolerance tests without clinical symptoms and/or hypoglycaemia.

**Figure 3:**
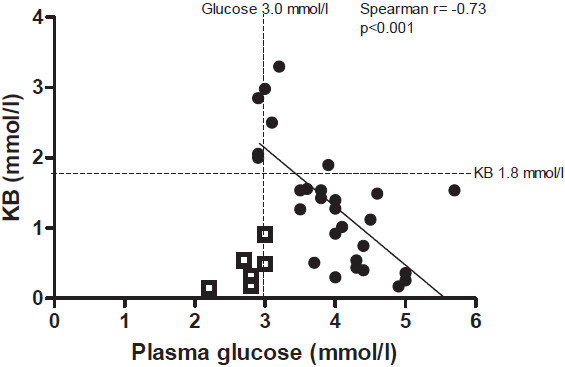
**Correlation between plasma glucose and KB during fasting tolerance test.** Relationship between blood glucose and KB concentrations is indicated. Case 5 is depicted in squares, as KB were low for glucose concentrations. The other cases (dots) are pooled.

Case 5 demonstrated normal fasting parameters after overnight fasting but hypoketosis after 18.5 hours. Low FFA concentrations that were not clearly increasing in time reflected a minimal role of mFAO under these circumstances in this subject, with subsequent low concentrations of KB (squares in Figure 3) [10,11].

Case 7 and case 9 also showed blood glucose concentrations that were below p10 for age and duration of fasting after 15 hours of fasting. FFA/KB ratios were at p10 for age and duration of fasting, with increasing concentrations of FFA and KB around p90. All other included subjects had KB concentrations that corresponded to blood glucose concentrations upon fasting (dots in Figure 3C).

A relationship between biochemical response to fasting and residual MCAD enzyme activity with either substrate could not be identified.

During fasting, C8:0 concentrations increased in time. Additionally, observed concentrations during fasting were considerably higher when compared to the concentrations that were seen during regular visits to the outpatient clinic (median during follow-up 0.6 umol/l; median after 15 h fasting 3.2 umol/l, p = 0.07). C8:0 concentrations during fasting did not correlate with concentrations found in the initial NBS test.

The C8:0/C2:0 and C8:0/C10:0 ratios during fasting were slightly higher than the ratios that were identified upon NBS and during regular visits to the outpatient clinic (Table 1). During the fasting tolerance test the ratios remained stable in time in all subjects [16].

In case 8, longer-chain acylcarnitines accumulated in time, besides medium-chain acylcarnitines (acylcarnitines after 18.5 hours of fasting: C12:0 0.60 μmol/l, C14:0 0.23 μmol/l, C14:1 0.75 μmol/l).

Prolonged fasting was associated with increasingly abnormal excretion patterns of organic acids, with *N-*hexanoylglycine, and dicarboxylic acids in the urine of all subjects with variant *ACADM* genotypes during fasting, as opposed to the observations during regular visits to the outpatient clinic, when few or no abnormal metabolites were be detected.

### PPA loading test

The fasting tests were combined with a PPA loading test. After oral administration of PPA, no phenylpropionylglycine (PP-glycine) or trace amounts were excreted in the urine, together with large amounts of hippuric acid.

## Discussion

Since the introduction of NBS for MCAD deficiency, a new subgroup of newborns has been identified with variant *ACADM* genotypes that have not been seen before in clinically ascertained patients with classical *ACADM* genotypes. It remains unclear whether subjects with these variant *ACADM* genotypes are at risk for the development of a clinical phenotype. Prevention of prolonged fasting was found to be debatable when MCAD enzyme activities >10% were measured with PP-CoA [2]. In the current study, additional support was provided to abandon the advice on prevention of prolonged fasting under normal conditions in subjects with residual MCAD enzyme activities >10%. All included subjects could tolerate an overnight controlled fasting tolerance test for at least 15 hours under healthy conditions. An additional PPA loading test determined *in vivo* residual MCAD enzyme activity. These functional tests were performed after the age of 6 months in all cases, when weaning naturally occurs and PPA loading tests can be performed reliably [14].

Several factors cause diversity between the subjects with variant *ACADM* genotypes that have been identified since introduction of NBS. Firstly, different *ACADM* genotypes are represented in this group. Secondly, the NBS protocol has been adapted in 2007, and has thus not been similar for all included subjects. Thirdly, treatment in the newborn period has not been the same in all subjects in this group. Identification of affected newborn siblings of probands was associated with dietary interventions from birth onwards in this group, instead of starting after positive NBS results.

Interestingly, two cases (case 3 and case 5) were identified upon NBS with C8:0 concentrations below the current cut-off concentration of 0.50 μmol/l, despite *ACADM* mutations on both alleles. Case 3 was identified upon family screening and case 5 during the pilot NBS program. With the elevation of the C8:0 cut-off concentration in 2007, the national criteria for subjects who should be regarded “patients” have also been adapted. However, due to family screening we still identify subjects with *ACADM* mutations on both alleles, but C8:0 concentrations below the cut-off value. How can we interpret these observations? C8:0 concentrations in newborns can theo-retically be influenced by nutritional state [5], prematurity and/or very low birth weight [17], heterozygosity for the c.985A>G mutation [18], and the time at which NBS is performed [19]. In our cohort, nutritional state can have affected NBS C8:0 concentrations, as these concentrations clearly correlated with percentage of maximum weight loss in the neonatal phase. Except for case 3, all subjects with a variant *ACADM* genotype in our cohort were firstborns who were breastfed. With breastfeeding caloric intake cannot be monitored, as opposed to during formula feeding. Especially in firstborns onset of lactation can be delayed, leading to suboptimal food intake in the first days of life [20]. Catabolism and subsequent increase in C8:0 concentrations can result from this. In case 3, C8:0 concentrations may have remained <0.50 μmol/l due to the positive family history and subsequent frequent feeding regimen that was started at birth.

Two subjects in our study (case 8 and case 9) displayed only one *ACADM* mutation after sequencing of the *ACADM* gene. Why have these subjects been identified upon NBS, as heterozygotes are usually not identified with the current C8:0 cut-off concentration [18]? Case 8 and case 9 were not considered normal false-positives, and where included in the cohort based on the combination of (1) persisting abnormal plasma and urinary metabolites, and (2) residual MCAD enzyme activity determined with C6-CoA. Results from fasting tolerance tests were remarkable in both subjects. In case 8, acylcarnitines with increasing chain-lengths up to C16:0 accumulated upon fasting. The C8:0 concentration increased considerably to a maximum of 4.7 μmol/l, whereas the C8:0/C10:0 remained <2.5. Theoretically, these findings may indicate another phenotype-modifying mutation on a gene that plays a role in mFAO, a phenomenon known as synergistic heterozygosity [21,22]. Vockley *et al.* described the concept of synergistic heterozygosity, i.e. multiple partial defects in more than one metabolic pathway, leading to clinical symptoms that correlate with the affected pathways [22]. In combination with the classical c.985A>G *ACADM* mutation, theoretical candidate genes to cause accumulation of acylcarnitines with medium and long chain-length are *ETFA* (HGNC:3481), *ETFB* (HGNC:3482), *ETFDH* (HGNC:3483), *ACADS* (HGNC:90) or *ACADVL* (HGNC:92) especially during episodes with increased catabolic stress such as during prolonged fasting. In case 8, C14:1 concentrations increased to a maximum of 0.75 μmol/l upon prolonged fasting, with a corresponding C14:1/C16:0 ratio of 4.4. The interpretation of these findings is complicated as, currently, no paediatric reference values for acylcarnitines during prolonged fasting are available. In case 9, the *in vivo* and *in vitro* observations might be explained by other molecular mechanisms (e.g. deep-intronic mutations, mutations in the promoter region, and deletion/duplication mutations) that can be missed upon gene sequencing, next to synergistic heterozygosity [23,24].

We observed a normal fasting tolerance in subjects with variant *ACADM* genotypes and residual MCAD enzyme activities >10% under normal conditions after the age of 6 months. Based on these results, the need to prescribe a standard late evening meal in these subjects is debatable. PPA loading tests cannot be reliably performed before the age of 6 months [14]. As PPA loading tests were in this study combined with the fasting tolerance tests, no data are currently available on fasting tolerance in subjects with variant *ACADM* genotypes and residual MCAD enzyme activities >10% before the age of 6 months. It is well known that even patients with classical *ACADM* genotypes can tolerate overnight fasting already at a young age [6,25]. Besides, stable isotope studies demonstrated normal FFA and KB metabolism in patients with classical *ACADM* genotypes [26,27]. Administration of a late evening meal has several disadvantages: First of all, it can be considered a burden for both parents and the child, especially in the first year of life when the maximum duration of fasting is 6–8 hours [28]. Additionally, administration of a late evening meal increases the risks on overfeeding and dental caries [6,29]. In our opinion, abovementioned arguments, combined with the current data, justify abolishing a standard late evening meal in subjects with variant *ACADM* genotypes and residual MCAD enzyme activities >10% after the age of 6 months. *In vivo* functional tests can facilitate development of individualized patient-based guidelines for follow-up reliably from the age of 6 months. However, until the pathophysiology of MCAD deficiency and its variants is unravelled, an emergency regimen and parental instructions remain indispensable in the follow-up of all subjects with a positive NBS for MCAD deficiency.

## Conclusions

Variant *ACADM* genotypes with residual MCAD enzyme activities >10% *in vitro* are associated with normal residual MCAD enzyme activities *in vivo* (defined by PPA loading) and normal fasting tolerance. The general advice to prevent prolonged fasting can be abolished in subjects with residual MCAD enzyme activities >10% after the age of 6 months. However, an emergency regimen and parental instructions remain necessary in all subjects with MCAD deficiency, regardless of residual MCAD enzyme activity, at least until the pathophysiology of MCAD deficiency has been elucidated.

## Abbreviations

ACADM: Gene encoding medium-chain acyl-CoA dehydrogenase; ACADVL: Gene encoding very long-chain acyl-CoA dehydrogenase; C2:0: Acetylcarnitine; C4-CoA: Butyryl-CoA; C6-CoA: Hexanoyl-CoA; ETF: Electron transfer flavoprotein; C8:0: Octanoylcarnitine; C10:0: Decanoylcarnitine; C10:1: Decenoylcarnitine; C14:1: Tetradecenoylcarnitine; C16:0: Palmitoylcarnitine; C8:0/C2:0: Ratio between octanoylcarntine and acetylcarnitine; C8:0/C10:0: Ratio between octanoylcarnitine and decanoylcarnitine; FFA: Free fatty acids; KB: Ketone bodies; MCAD: Medium-chain acy-CoA dehydrogenase; mFAO: Mitochondrial fatty acid oxidation; NBS: Newborn bloodspot screening; PPA: Phenylpropionic acid; PP-CoA: Phenylpropionyl-CoA; PP-Glycine: Phenylpropionylglycine.

## Competing interests

The authors declare that they have no competing interest.

## Authors’ contributions

CML Touw participated in data collection, analysis and interpretation, generation of the figures, in writing of the manuscript, and approved of the final version. KE Niezen-Koning participated in data collection, and approved of the final version. C Bosgraaf-de Boer participated in laboratory analyses and data collection, and approved of the final version. A Gerding participated in laboratory analyses and data collection, and approved of the final version. GPA Smit, DJ Reijngoud and TGJ Derks conceived the study, participated in writing the manuscript, and approved of the final version.
